# Extracellular Cysteine in Connexins: Role as Redox Sensors

**DOI:** 10.3389/fphys.2016.00001

**Published:** 2016-01-28

**Authors:** Mauricio A. Retamal, Isaac E. García, Bernardo I. Pinto, Amaury Pupo, David Báez, Jimmy Stehberg, Rodrigo Del Rio, Carlos González

**Affiliations:** ^1^Facultad de Medicina, Centro de Fisiología Celular e Integrativa, Clínica Alemana Universidad del DesarrolloSantiago, Chile; ^2^Facultad de Ciencias, Centro Interdisciplinario de Neurociencias de Valparaíso, Instituto de Neurociencias, Universidad de ValparaísoValparaíso, Chile; ^3^Laboratorio de Neurobiología, Centro de Investigaciones Biomédicas, Universidad Andres BelloSantiago, Chile; ^4^Laboratory of Cardiorespiratory Control, Center for Biomedical Research, Universidad Autónoma de ChileSantiago, Chile; ^5^Dirección de Investigación, Universidad Científica del SurLima, Perú

**Keywords:** connexins, hemichannels, redox potential, gap junction channels, post-translational modification, gaseous transmitters

## Abstract

Connexin-based channels comprise hemichannels and gap junction channels. The opening of hemichannels allow for the flux of ions and molecules from the extracellular space into the cell and vice versa. Similarly, the opening of gap junction channels permits the diffusional exchange of ions and molecules between the cytoplasm and contacting cells. The controlled opening of hemichannels has been associated with several physiological cellular processes; thereby unregulated hemichannel activity may induce loss of cellular homeostasis and cell death. Hemichannel activity can be regulated through several mechanisms, such as phosphorylation, divalent cations and changes in membrane potential. Additionally, it was recently postulated that redox molecules could modify hemichannels properties *in vitro*. However, the molecular mechanism by which redox molecules interact with hemichannels is poorly understood. In this work, we discuss the current knowledge on connexin redox regulation and we propose the hypothesis that extracellular cysteines could be important for sensing changes in redox potential. Future studies on this topic will offer new insight into hemichannel function, thereby expanding the understanding of the contribution of hemichannels to disease progression.

## Introduction

**Figure 1:**
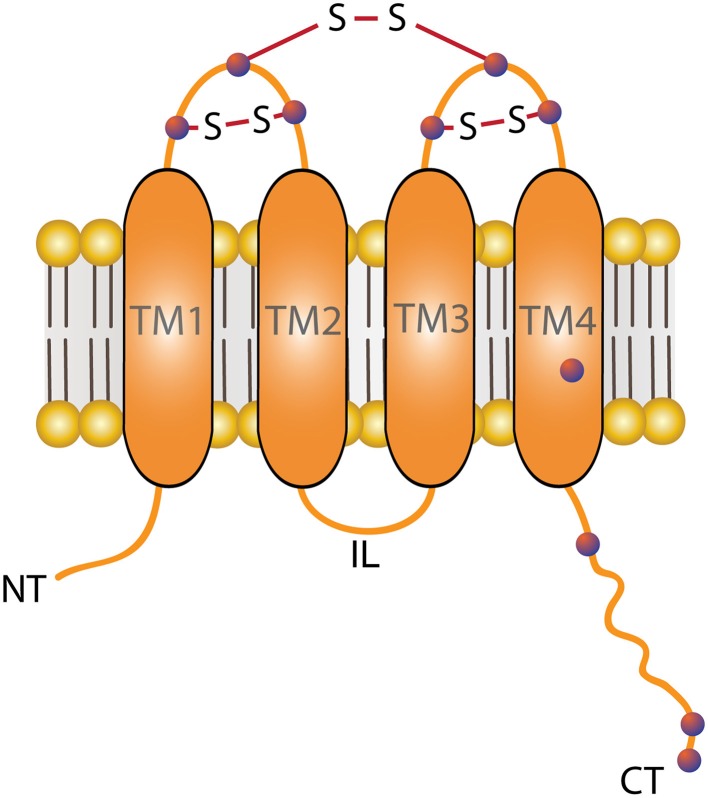
**Plasma membrane topology of Connexin**. Connexin proteins are formed by four transmembrane domains (TM1-TM4), three intracellular segments; the N-termini (NT), an intracellular loop (IL) and the C-termini (CT) and two extracellular loops each containing three Cys (purple dots), which can form disulphide bonds (-S-S-) between Cys located in different loops. However, biochemical evidence suggests that formation of disulphide bond intraloops cannot be excluded (Foote et al., 1998). Connexins also have some free Cys in their TM4 and/or in the CT segments.

Connexins are a large family of transmembrane proteins involved in cellular communication. In humans, there are 21 genes encoding for connexins (Söhl and Willecke, 2003). These proteins are named based on their predicted molecular weight, connexin23 (Cx23, ~23 kDa) being the smallest, and Cx62 (62 kDa) the largest (reviewed in Sáez et al., 2003). Despite the different amino acid sequences among connexin isoforms, they share similar plasma membrane topology, which include the N- and C-termini (NT and CT respectively) being oriented toward the cytoplasm, four transmembrane domains (TM1-TM4), one intracellular loop (IL), and two extracellular loops (EL1 and EL2) (Figure 1; Rahman et al., 1993; Yeager and Nicholson, 1996). Oligomerization of six connexin subunits forms a channel known as hemichannel. Growing evidence support the role of hemichannels as a route to pass ions and signaling molecules that participates in paracrine and autocrine signaling in normal and pathological conditions.

An important milestone in the structural and functional understanding of connexins came with the 3.5 Angstrom resolution of the X-ray crystal structure of Cx26 (Maeda et al., 2009). In terms of gating regulation, the structure confirms previous reports proposing that the NT of Cx26 bends inside the channel pore to form a plug (Purnick et al., 2000; Oshima et al., 2007; Maeda et al., 2009). Thereby the NT of connexins partakes in determining the gating properties of hemichannels (Kronengold et al., 2012). However, little is known about the tertiary conformational structure of the Cx26-CT, which was not resolved in Maeda and colleagues study, suggesting that the CT may not have a defined conformation (Maeda et al., 2009). This is in agreement with the nuclear magnetic resonance studies of Sorgen and colleagues, who reported that this segment has a random coil structure with two α-helices (Sorgen et al., 2004).

In the last two decades, the role of hemichannels in cell function has been studied in more detail. Paul and colleagues were the first reporting the presence of hemichannels (Cx46) at the plasma membrane. Moreover, they showed that the opening of Cx46 hemichannels could induce cell death (Paul et al., 1991). Thus, for several years, it was thought that hemichannels must be closed to prevent cell lysis, detracting their possible physiological relevance. However, current experimental evidence show that hemichannels do open with low open probability (Contreras et al., 2003), but sufficient to regulate important physiological processes (for more details see Chandrasekhar and Bera, 2012; Kar et al., 2012; Cheung et al., 2014). In agreement with previous observations, prolonged and/or massive opening of hemichannels is linked to cell damage by leakage of metabolites and ionic imbalance (Retamal et al., 2015). Given their importance for physiological functions and cell survival, further studies are needed in order to understand the regulatory mechanisms that control their gating and permeability of hemichannels.

## Extracellular cysteines: Role for GJC formation

Early in the nineties, it was shown that the formation of Cx32 GJC depends on the redox status of extracellular cysteines (Cys) (Dahl et al., 1991). In this work, the authors found that extracellular application of high concentrations (10–14 mM) of reducing agents, such as 2-mercaptoethanol and dithiothreitol (DTT) decreases GJC currents as consequence of an impaired GJC formation. In contrast, low concentrations of these agents (0.01–0.14 mM) promote an increase of GJC currents. This finding was attributed to a faster GJC formation rather than a rise of their open probability (Dahl et al., 1991). The redox modulation was reversible since GJC currents were restored after washout. Interestingly, the addition of the impermeable reducing agent -maleimide butyryl biocitin (MBB)- on 2-mercaptoethanol treated oocytes, induces a greater reduction of GJC currents. Given that the binding of MBB is irreversible, the authors suggested that MBB blocks the *novo* formation of GJCs, possibly by a steric hindrance that could block the disulphide bond formation, hence preventing hemichannels to dock properly. Furthermore, the exchange of any of the six extracellular Cys into a serine resulted in dysfunctional GJCs.

All together, these data suggests that extracellular Cys are critical for GJC formation and forms disulphide bonds under normal conditions (Dahl et al., 1991). In this regard, using trypsin-digested rat hepatocytes expressing Cx32, Rahman and colleagues determined that all six extracellular Cys form three disulfide bonds (Rahman et al., 1993). In particular, it was suggested that Cys from one extracellular loop form disulfide bonds with those located at the following loop (Rahman and Evans, 1991; Foote et al., 1998; Figure 1). Moreover, in Maeda's work, it was shown that intramolecular disulphide bonds are formed between loops rather than intra-loop bonds (Maeda et al., 2009). However, the possible formation of intra-loop disulphide bonds (Foote et al., 1998) cannot be ruled out. We would like to point out that in all these experiments the authors considered the protein contained in the whole cell lysate, therefore it is unknown if a small pool of hemichannels with free extracellular Cys (-SH) are present in physiological conditions.

## The extracellular cysteines: Role in hemichannel function

As mentioned before, the presence of six extracellular Cys per subunit is a general rule for connexins, except for Cx23. This connexin has four extracellular Cys due to the absence of the second Cys pair present in each extracellular loop (Iovine et al., 2008). This has been reported that all extracellular Cys are required for GJC formation (Dahl et al., 1991), nonetheless Cx23 form functional hemichannels and GJCs (Iovine et al., 2008). This data suggests that the complete set of six extracellular Cys is not critical for GJCs and hemichannel function. Later, using *Xenopus laevis* oocytes expressing an extracellular-Cys-deficient Cx43 (named as CL Cx43D2), it was shown that this connexin-mutant does not form GJCs, however was able to form functional hemichannels (Bao et al., 2004). Moreover, both the mutant and wild type Cx43 hemichannels were regulated in the same way by PKC. These data suggests that although extracellular disulphide bonds are important for GJCs formation, they are not crucial for functional hemichannels formation. Congruent with this idea, using dye uptake and electrophysiological recordings, Cx43eGFP hemichannels were shown to significantly increase their activity after exposure to 10 mM DTT (Retamal et al., 2007), a concentration expected to disrupt disulphide bonds. Based on these results, we can assume that hemichannel function increases when one or more disulphide bonds are disrupted. However, this assumption may not be true for all connexin isoforms since it has been reported that Cx37 lacking extracellular Cys (Cx37-C6A), localizes at the plasma membrane, but does not form functional hemichannels or GJCs (Good et al., 2014).

## Extracellular vs. intracellular connexin cysteines: Their role as redox sensors

**Figure 2:**
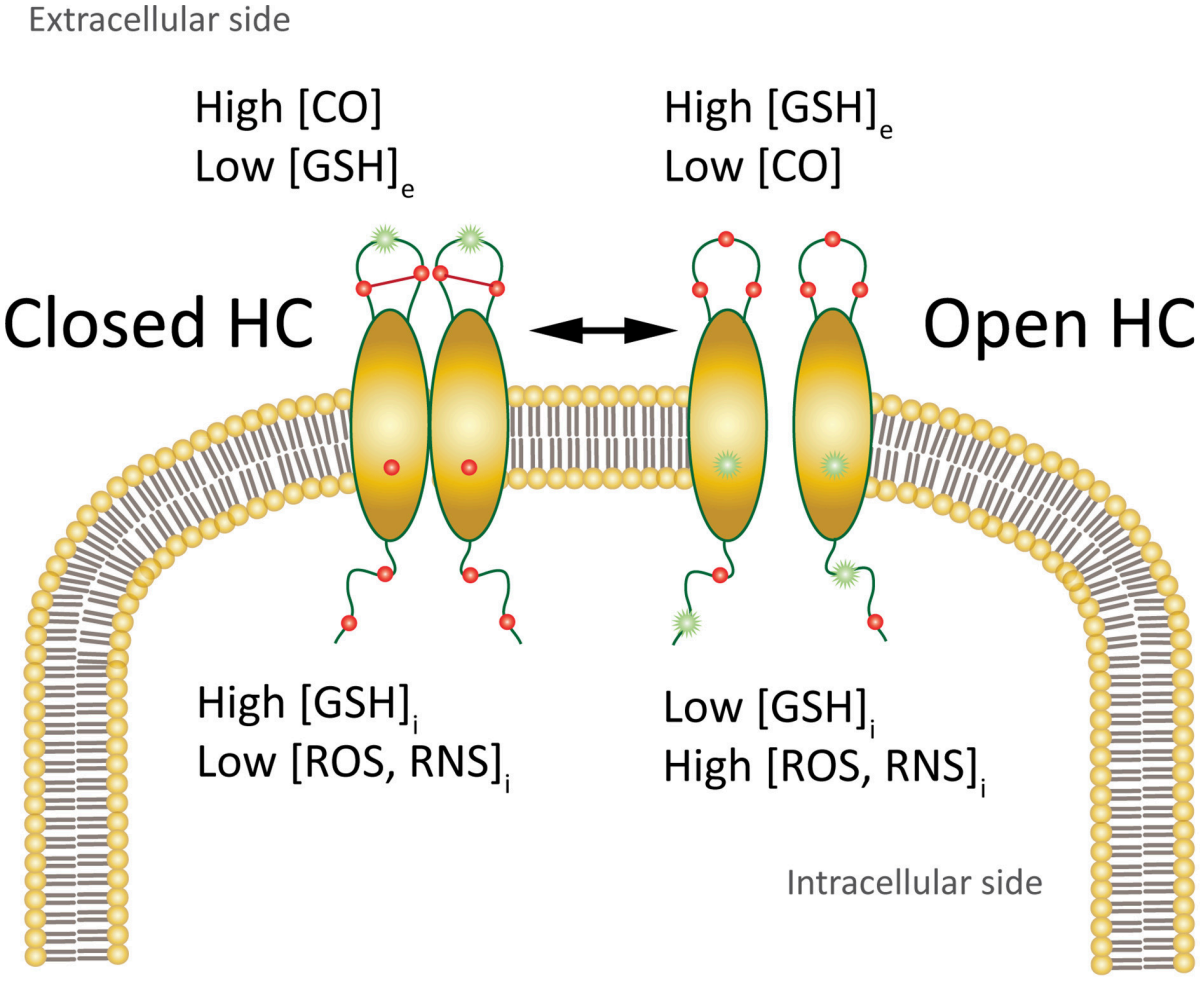
**Hypothetic Model**. When Cys (Red dots) located at CT and/or TM4 are in a reduced status, hemichannels are mostly closed. Therefore, under physiological conditions when the concentration of reduced gluthathion (GSH) is high and free radicals (ROS and RNS) are low, hemichannels will exhibit a low open probability, as shown by Contreras and co-workers (Contreras et al., 2003) and many others. On the contrary, when intracellular or TM4 Cys are oxidized (e.g., S-nitrosylated) changes in the hemichannels properties are observed. In the case of Cx43, this oxidation is associated to increase hemichannel activity (Retamal et al., 2006). Interestingly, current evidence allows us to suggest that there is a pool of hemichannels at the plasma membrane with reduced extracellular Cys, which allows them to open frequently. When these reduced Cys become oxidized (e.g., forming disulfide bonds or carbonylated; León-Paravic et al., 2014), hemichannels become mostly closed. Green dots represent Cys oxidized by free radicals such as ROS, RNS or carbon monoxide (CO).

**Figure 3:**
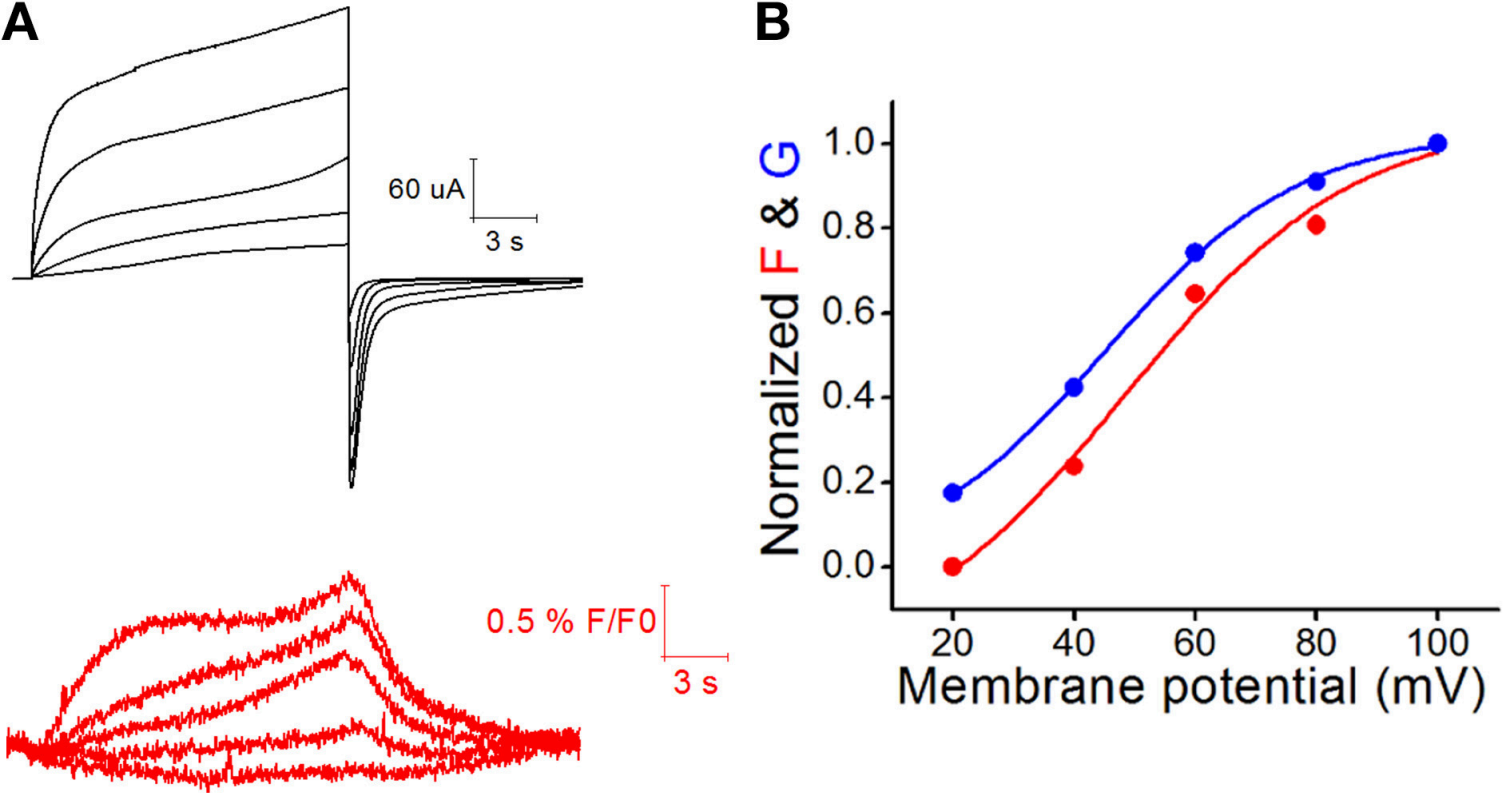
**Voltage-clamp fluorometry recording of Cx46 hemichannels currents expressed in ***X. laevis*** oocytes**. The voltage clamp fluorometry (VCF) is a technique in which fluorophores (e.g., TAMRA) that covalently binds the thiol groups of cysteines (native or introduced by site-directed mutagenesis) located in ion channel domains involved in voltage sensing. VCF allow the simultaneous recording of currents (patch clamp) and fluorescence. Under voltage stimulation, the channel undergoes conformational changes and the movement of the molecular sensor is evidenced by change in the fluorescence emission intensity. For more detail see Zheng (2006). Oocytes were injected with cRNA to induce Cx46 expression. After 24 h post-injection oocytes were bathed in recording solution containing 50 μM of TAMRA, a non-permeable molecule that specifically binds to free Cys and emits fluorescence in response to changes in its environment. **(A)** Hemichannel currents (upper trace) where recorded in a dual electrode whole cell voltage clamp configuration. Oocytes were held at −60 mV and 20 mV depolarization step were applied from +20 to +100 mV. Changes in fluorescence (lower trace) were simultaneously recorded. **(B)** Normalized fluorescence and hemichannel current at different voltages showing that both recordings present almost the same profile of changes, strongly suggesting that the fluorescence recorded correspond to changes in Cx46 hemichannel structure induced by plasma membrane voltage. Our preliminary data suggest that there is a pool of hemichannels that present free Cys in their extracellular loops, because TAMRA was able to bind it and also corroborate that extracellular loops move in response to changes in plasma membrane voltage (Verselis et al., 2009).

It is known that nitric oxide (NO) induces the opening of Cx43 hemichannel in astrocytes (Retamal et al., 2006). Indeed, NO promotes the S-nitrosylation of the Cx43 intracellular Cys271 in an *ex vivo* model of blood vessel wall (Straub et al., 2011). Additionally, hemichannels formed by Cx46 are also sensitive to NO, and appear to be mediated by some type of intracellular Cys- oxidation, because the NO donor GSNO does not modify the functional properties of Cx46 hemichannels when intracellular Cys are mutated to Ala (Retamal et al., 2009). Thus, we hypothesized that both intracellular and extracellular Cys control some hemichannel properties by changing their redox status (Figure 2). Accordingly, carbon monoxide (CO), which is another gaseous transmitter that modulates the redox status in cells, induced an important current reduction of Cx46 hemichannels (León-Paravic et al., 2014). This effect was observed in *Xenopus laevis* oocytes expressing a Cx46 mutant lacking intracellular Cys (Cx46C3A), but was absent in oocytes expressing a Cx46 Cys-lacking mutant (Cx46CL), suggesting that extracellular Cys is/are important(s) for the inhibition of Cx46 hemichannels induced by CO. Moreover, this inhibition was fully reverted by reduced glutathione (5 mM GSH), which is not permeable to the plasma membrane. Considering these data, it is possible to postulate that, the redox status of connexin extracellular Cys may be more dynamic than previously thought and that the presence of a pool of hemichannels with one or more Cys in -SH status is plausible under physiological conditions. Here we propose that Cys in -SH status at the extracellular loop of hemichannels may act as redox sensors, taking part in the regulation of their gating properties. To support this hypothesis, we measured Cx46 hemichannels fluorescence/currents using Voltage Clamp Fluorometry with a plasma membrane-impermeable fluorophore (TAMRA) that binds to extracellular –SH groups. The fluorescent emission under electrical stimulation was used to estimate conformational changes in the extracellular loops (Figure 3). Given that the fluorophore only binds to extracellular reduced Cys, it is possible to suggest that under control conditions there are Cx46 extracellular Cys in reduced configuration. In conclusion, NO seems to act only through oxidation of intracellular Cys, while CO seems to act through oxidation of extracellular –SH. Further exploring these hypotheses will increase our current knowledge of this potential hemichannel regulatory mechanism.

## Possible implications of hemichannel redox modulation

It is well known that the intracellular redox status is controlled by several mechanisms including the balance between reduced (GSH) vs. oxidized (GSSH) glutathione concentrations (normally denoted as GSH/GSSG ratio), and the action of reducing enzymes such as peroxidases, catalases, dismutases, thioredoxins, and peroxiredoxins among others (Wadhwa and Mumper, 2013). The balance between GSH and GSSG is the best indicator for the intracellular redox status because of the ability of GSH to either reduce oxidized Cys by itself (Pastore et al., 2003), or regulate the activity of reducing enzymes (Circu and Aw, 2012).

There are a number of evidence showing the importance of redox potential for different cellular processes. However reports regarding *in vivo* redox-regulation of hemichannels are scarce. We next will give some examples of cellular processes in which intracellular and extracellular redox potential could modulate the hemichannel activity.

### Astrocytes

Astrocytes are the most abundant type of brain cells and have critical roles in brain function, such as the modulation of neuronal synapses through the release of gliotransmitters (Del Rio et al., 2015), controlling neuronal metabolism (Falkowska et al., 2015), and neuronal redox status (Wilson, 1997; Lane and Lawen, 2009) among other processes. Connexin hemichannels are involved in many of these astrocyte's functions (Orellana and Stehberg, 2014). In this context, Stehberg and co-workers have shown that gliotransmitter release mediated by Cx43-hemichannels expressed in astrocytes of the lateral amygdala, contribute to the control of the neuronal synaptic transmission (Stehberg et al., 2012). Likewise, Chever and colleges found that astrocytes release ATP through Cx43 hemichannels which in turn regulates basal synaptic transmission of hippocampal CA1 pyramidal neurons (Chever et al., 2014). Interestingly, studies in hippocampal co-cultures, revealed a NO production in astrocytes induced by extracellular ATP. The NO can diffuse to neighboring neurons causing neuronal synapsis depression (Mehta et al., 2008). Accordingly, the diffusion of NO through the plasma membrane burst when Cx43 hemichannels open (Figueroa et al., 2013). Bearing in mind that Cx43 hemichannels are S-nitrosylated by NO in astrocytes (Retamal et al., 2006), we could suggest this mechanism as a potential synaptic transmission modulator.

Changes in intracellular redox potential can alter the extracellular redox status and these changes are correlated with cellular processes such as proliferation, differentiation, and apoptosis (Banerjee, 2012). There are chemical and enzymatic mechanisms, that control the redox status of the extracellular space. In terms of chemical control, it is known that GSH can be found in the extracellular space at concentrations (~500 μM) lower than those found in intracellular compartments (3–10 mM) (Cantin et al., 1990; Ottaviano et al., 2008). In the brain, one of the pathways for GSH release from astrocytes is through the opening of Cx43 hemichannels (Rana and Dringen, 2007). Extracellular application of 5 mM DTT increases Cx43 hemichannel activity in HeLa cells (Retamal et al., 2007), suggesting that in astrocytes, could exist a positive feedback between the extracellular GSH concentration and the activity of Cx43 hemichannels. Moreover, Tong and colleagues, recently showed that Cx26 hemichannels are permeable to GSH, which was able to reduce Cys groups placed in a segment located in the transition between TM4-EL1 (Tong et al., 2015). This, data suggest that the GSH could contribute to reduce the disulfide bridges between extracellular loops, increasing their open probability even more. However, this process must require a fine regulation since prolonged hemichannel opening could lead to cellular damage due to a massive ATP and glutamate release in astrocytes and/or neurons (Orellana et al., 2011; Retamal et al., 2015). But DTT at concentrations below 1 mM does not induce any modification in Cx43 hemichannel activity in HeLa cells (Retamal et al., 2007). This suggests that, although the GSH concentration may be higher in some extracellular micro-domains; extracellular GSH may not be able to modulate hemichannel activity by reducing their disulfide bonds *in vivo*. Therefore, it is more likely that the enzymatic system found in the extracellular space has more influence in the redox status of the extracellular protein domains. Likewise, the glutathione peroxidase 3 (GPX3), disulphide isomerase (PDI), peroxiredoxin (PRx) and thioredoxin (TRx1), are well known regulators of the extracellular redox status (Hawkes and Alkan, 2010; Griffiths et al., 2014). Under physiological conditions it has been shown that extracellular TRx regulates the redox status of the membrane receptor CD30 in lymphocytes (Schwertassek et al., 2007). As previously reported, TRx is released from several normal and neoplastic cells (Matsuo and Yodoi, 2013), additionally TRx's extracellular concentration is increased in pathological conditions (Matsuo and Yodoi, 2013). Once in the extracellular space, TRx is able to reduce disulfide bonds to -SHs recovering its reducing power; and oxidized TRx is reduced by the NADPH dependent flavoenzyme thioredoxin reductase (TRxR) (Matsuo and Yodoi, 2013). Until now, no direct effect of TRx or any other enzyme relate to redox control on astrocytic hemichannels has been found, but it has been suggested that at least Trx may participate in the decarbonylation process observed in Cx46 after carbon monoxide exposure (León-Paravic et al., 2014). Therefore, it is evident that more research is needed about the role of redox control upon hemichannels under physiological conditions in brains cells.

### Vascular system

The main types of cells present in the vascular system are the endothelial cells and smooth muscle cells. Endothelial cells produce NO by means of endothelial nitric oxide synthase (eNOS) activity. Then, NO diffuses into smooth muscle cells inducing their relaxation. Because NO is a highly reactive molecule, to modify hemichannel properties eNOS would be expected to be in very close proximity to the Cxs present in endothelial cells Cx37, Cx40, and Cx43. Indeed, by means of coimmunoprecipitation and confocal studies, it was observed that Cx37 and Cx40 interact with this NO producing enzyme (Pfenniger et al., 2010; Le Gal et al., 2015; Meens et al., 2015). The S-nitrosylation of Cx43 in endothelial cells decreases the passage of IP3 between endothelial cells to smooth muscle cells via GJCs (Straub et al., 2011). In this work the effect of NO on hemichannels was not reported, however, NO seems to need functional hemichannels to cross the plasma membrane (Figueroa et al., 2013), and moreover, NO induce the S-nitrosylation of Cx37 and Cx40 (Figueroa et al., 2013). This suggest that NO autoregulate its pass through the plasma membrane and also may control hemichannel permeability in endothelial cells.

### Retina

The retina is a tissue that enables the transduction of light into electrical signals that are processed by the brain (Mannu, 2014). In response to light amacrine cells release dopamine and NO (Koistinaho et al., 1993) which through the activation of a NO/cGMP/PKG dependent pathway induce the inhibition of GJC of horizontal cells (Miyachi et al., 1990). Interestingly, it has been described an ephaptic communication between cones and horizontal cells through the opening of Cx26 hemichannels present in horizontal cells (Vroman et al., 2013). In spite of Cx26 having several Cys that could be modulated by NO or other redox signaling molecules, there is no evidence of their modification by redox potential. Thus, it's possible to suggest that Cx26 hemichannels in the retina could be controlled by NO, and thus participate in light processing.

### Kidney

Several connexins are expressed in the kydney, such as Cx26, Cx30, Cx30.3, Cx37, Cx40, Cx43, and Cx45 (Hanner et al., 2010). Regarding the role of hemichannels in this system, it has been reported that juxtaglomerular endothelial cells release ATP through Cx40 hemichannels, contributing to the propagation of purinergic and calcium signaling. The authors suggested that this phenomenon could be important for the control of glomerular filtration and renin release (Toma et al., 2008). As it has been probed that NO is important for the renin secretion from the macula densa (Castrop et al., 2004), it could be possible that NO through the S-nitrosylation of Cx40 may affect juxtaglomerular function. On the other hand, Cx30 hemichannels located in membrane of cells in the distal nephron play an important role in the control of pressure natriuresis by releasing ATP into the tubular fluid, which inhibits salt and water reabsorption (Sipos et al., 2009). Laminar flow inside of the renal tubes induce NO production from epithelial cells. In spite of that, the effect of NO on the tubular physiology is not clear; it could be possible that NO increase the activity of Cx30 hemichannels, which in turn will have a great impact in the water reabsorption. Extracellular GSH in the kidney is important for the mercury (Hg) tubular transport (Lash et al., 1998). This result support the idea that GSH is released in the kidney and has an important role in the Hg transport, therefore GSH could play role in hemichannel control along the renal tubules.

Several experiments are necessary to test the hypothesis above described. Therefore, we want to encourage to the scientific community to study the possible interaction of connexin hemichannels and enzymes related to redox control and how hemichannels are controlled by changes in GSH levels.

## Conclusion

Connexin hemichannel function is controlled by several molecular mechanisms, including phosphorylation, membrane potential, and protein-protein interaction, among others. Recently, it has been proposed that changes in redox potential may induce changes in hemichannels properties. However, the exact underlying molecular mechanism is still unclear. Based on the current knowledge and our preliminary results, we hypothesized that extracellular Cys reacts to changes in the redox potential. These modifications induce the opening of connexin hemichannels when extracellular Cys become reduced and close hemichannels when Cys become oxidized. Undoubtedly, more studies are necessary to identify the main redox molecule(s) responsible for connexin extracellular Cys oxidation under physiological and pathological conditions. In addition, it will be interesting to determine whether oxidant molecules act differentially through oxidation of intracellular or extracellular Cys. In conclusion, the extracellular redox status is dynamic and could be modulated by the GSH concentration as well as enzymes such as Trx in a physiological context. Under pathological conditions, both GSH and the extracellular enzyme content, changes; impairing the redox modulation of –SH and disulfide bond formation and it is likely that SH-related post-translational modification could be affected.

## Author contributions

MR, IG, BP, AP, DB, JS, RD, and CG, All of them wrote and edited this manuscript. MR and BP, Performed Electrophysiological experiments. MR, BP, DB, and CG, analyzed data. MR, IG, AP, DB, JS, RD, and CG, Wrote the paper. MR, IG, made figures.

## Funding

This work was partially funded by FONDECYT 1120214 and Anillo ACT-1104 (to MR), FONDECYT 3150634 (to IG), Conicyt doctoral fellowship (AP), and FONDECYT 1120802 (to CG). FONDECYT 1130724 (JS) and UNAB DI-603-14/N (JS). Conicyt PFCHA 2014 (BP). The Centro Interdisciplinario de Neurociencias de Valparaíso is a Chilean Millennium Institute (P09-022-F).

### Conflict of interest statement

The authors declare that the research was conducted in the absence of any commercial or financial relationships that could be construed as a potential conflict of interest.
